# A novel caspase 8 selective small molecule potentiates TRAIL-induced cell death

**DOI:** 10.1038/srep09893

**Published:** 2015-05-11

**Authors:** Octavian Bucur, Gabriel Gaidos, Achani Yatawara, Bodvael Pennarun, Chamila Rupasinghe, Jérémie Roux, Stefan Andrei, Bingqian Guo, Alexandra Panaitiu, Maria Pellegrini, Dale F. Mierke, Roya Khosravi-Far

**Affiliations:** 1 Department of Pathology, Beth Israel Deaconess Medical Center and Harvard Medical School, Boston, MA, USA; 2 Department of Chemistry, Dartmouth College, Hanover, NH, USA; 3 Department of Systems Biology, Harvard Medical School, Boston, MA, USA; 4 Institute of Biochemistry of the Romanian Academy, Bucharest, Romania; 5 Department of Biological and Biomedical Sciences Program, Harvard Medical School, Boston, MA, USA

## Abstract

Recombinant soluble TRAIL and agonistic antibodies against TRAIL receptors (DR4 and DR5) are currently being created for clinical cancer therapy, due to their selective killing of cancer cells and high safety characteristics. However, resistance to TRAIL and other targeted therapies is an important issue facing current cancer research field. An attractive strategy to sensitize resistant malignancies to TRAIL-induced cell death is the design of small molecules that target and promote caspase 8 activation. For the first time, we describe the discovery and characterization of a small molecule that directly binds caspase 8 and enhances its activation when combined with TRAIL, but not alone. The molecule was identified through an *in silico* chemical screen for compounds with affinity for the caspase 8 homodimer’s interface. The compound was experimentally validated to directly bind caspase 8, and to promote caspase 8 activation and cell death in single living cells or population of cells, upon TRAIL stimulation. Our approach is a proof-of-concept strategy leading to the discovery of a novel small molecule that not only stimulates TRAIL-induced apoptosis in cancer cells, but may also provide insights into the structure-function relationship of caspase 8 homodimers as putative targets in cancer.

Apoptotic cell death is a mean to eliminate unwanted or damaged cells^1^,^2^,^3^. Caspase 8 is a cysteine protease that initiates a cell death response mediated by a group of receptors of the tumor necrosis factor (TNF) superfamily, including TRAIL, commonly known as death receptors^1^,^4^. Dysregulation of the apoptotic process makes cancer cells resistant to TRAIL and conventional therapies. Apo2L/TRAIL is a cytokine known for its potential to selectively kill cancer cells and it is an important candidate for targeted cancer therapy. However, many cancer cells show resistance to TRAIL-induced apoptosis and efficient strategies for sensitization to TRAIL have to be developed^3^. The apoptosis process is triggered by recruitment of adaptor proteins, such as FAS-associated death domain (FADD), to the death receptors, which is followed by recruitment of caspase 8 pro-enzyme. This causes dimerization of caspase 8 resulting in its activation. This death receptor recruitment process also brings a variety of other proteins to the death-inducing signaling complex (DISC)^1^,^5^,^6^, which forms within biologically important and dynamic membrane domains named lipid rafts^7^.

Among the proteins recruited to the DISC is cellular FLICE-like inhibitory protein, long isoform (FLIP_L_)^8^. cFLIP_L_ is homologous to caspase 8 but has a pseudocaspase domain which lacks catalytic residues^9^. Caspase 8-cFLIP heterodimers are formed at the DISC, when FADD recruits both FLIP and caspase 8 to its cytoplasmic tails. Active caspase 8 generated from an efficient DISC can be inhibited by high levels of c-FLIP, antagonizing caspase 8 activation. Although cFLIP inhibits apoptotic activation of caspase 8, caspase 8-cFLIP heterodimer complexes intriguingly may possess catalytic activity^10^. *In vitro* and structural studies suggest that the formation of caspase 8-cFLIP heterodimers is favored over caspase 8 homodimers, as cFLIP has a greater affinity for pro-caspase 8 than for itself^10^. The crystal structure of the protease-like domain of cFLIP_L_ in complex with zymogen caspase 8 reveals insights into the favorable heterodimerization complex over the pro-caspase 8 homodimerization^11^. Heterodimerization constrains the active site of zymogen caspase 8 into a productive conformation, resulting in proteolysis of a number of local substrates such as RIP^12^, even in the absence of the interdomain autocleavage events that are necessary for stabilization and activity of caspase 8 homodimer^10^,^11^,^12^. Noteworthy, although cFLIP_L_ can initiate the first proteolytic cleavage of pro-caspase 8, it also prevents subsequent cleavage of caspase 8. Activation of caspase 8 induced by caspase 8-cFLIP heterodimer is different from activation occurring in the pro-caspase 8 homodimer, resulting only in partial activation of caspase 8, which is restricted to the cellular membrane^13^. Thus, pro-caspase 8 homodimers, resulting in an increase in the mature caspase 8 homodimers, are needed to induce fast and efficient apoptosis.

Numerous caspase inhibitors targeting the active sites of caspases have been developed. The pocket at the caspase dimerization interface of caspases has also been successfully targeted with the goal to identify novel inhibitors. Structural studies of the caspase 7 dimer interface using disulfide trapped inhibitors reversed the structural transition and induced a structural conformation that was almost indistinguishable from the zymogen form^14^. By using caspase 1, it has been shown that a general allosteric binding site for reversing the caspase activation with small molecules exists^15^. All of these inhibitors block the apoptotic process. However, instead of inhibiting caspases it would be of considerable medical interest to activate them and induce caspase-dependent cell death in cancer cells, tissue and tumors that are resistant to apoptosis.

We therefore developed and employed an innovative strategy to discover novel small molecules that would bind at the caspase 8 interface and stabilize the caspase 8 homodimers. The role of cleavage in caspase 8 activation is complex. Pro-caspase 8, an inactive precursor or zymogen, undergoes major structural transitions after auto- or trans-proteolysis, which results in interdomain cleavages in a defined order, yielding mature caspase 8, containing two large and two small subunits. Structural studies have shown that the β6-strand dimerization interface of mature caspase 8/zymogen caspase 8 homodimers have reduced hydrogen bonding capacity compared to zymogen caspase 8-cFLIP heterodimer^11^. The formation of the zymogen caspase 8 homodimer is further obstructed by the presence of the L2 loops^11^. If pro-caspase 8 homodimerization could be favored by additional stabilization or interaction, it would result in a productive conformation that is able to initiate faster and more efficient auto- or trans-proteolysis, resulting in mature caspase 8 homodimers. This could result in enhanced initiator caspase activation and caspase 8 activity. In order to identify small molecules with affinity for the caspase 8 homodimeric interface, we carried out a virtual screening using a library of 3,000 small molecules. The best hits were experimentally validated in a series of binding and activation assays. One of the small molecules identified demonstrated activity both in cancer cells that are resistant and cancer cells that are sensitive to caspase-dependent cell death induced by TRAIL. These results taken in sum provide insight into the structure-function relationship of caspase 8 homodimers as a putative target in drug strategies for targeting cancer.

## Materials and Methods

### *In silico* identification of the small molecules

The Virtual Screening (VS) was carried out using AutoDock Suite 4.0.1^16^. In short, the protease-like domain of pro-caspase 8 (PDB ID: chain B of 3H11) was used as the target receptor. A library of 3,000 small molecules (Life Chemicals Inc.) was targeted against pro-caspase 8. Hydrogen atoms were included into all *3D* coordinates of the receptor and the ligands. Atomic charges were set for the ligands and the receptor using AutoDock Tools 1.5.4. All bonds of the ligands were considered as flexible and the receptor was kept rigid. A grid center was introduced, which functioned as the starting point in the search space. AutoGrid 4.0.1 was carried out to generate the map files for the flexible atoms. The docking parameter file (DPF) was defined with the Genetic algorithms (either *Lamarkian genetic algorithm* or *Hybrid genetic algorithm*), maximum number of energy evaluations, initial population, number of runs and maximum RMS tolerance for conformational cluster analysis. DPF was generated using the map files and 3D coordinates files with atom charges. AutoDock 4.0.1 was applied for the ligand library. During the search process, binding energies were calculated considering non-bonded interactions (Van-de Walls and electrostatic interactions), H-bonding, desolvation and torsional energy of the complexes. Finally, the binding results were sorted according to the minimum binding energy for the best pose and the conformation of the ligand on the binding site *i.e.,* the lowest energy conformation from the largest cluster chosen as representative. Top hits were selected for further molecular and cellular experimental analysis.

### Molecular modeling of the Caspase 8 / Compound 2 complex

Homology model for zymogen caspase 8 monomer 1 was built using PDB ID: 3H11_chain B as the template (89% sequence identity) using SWISS MODEL^16^,^17^. Compound 2 was docked into the dimer interface of monomer 1 using AutoDock 4.01^18^ to identify the mode of binding. The caspase 8 homodimer (with monomers 1 & 2) was generated using the X-ray structure 3H11 as a guide for the placement of monomer 2. The resulting complex was then relaxed by energy minimization using s*teepest descent* followed by *conjugate gradient* for 400 steps using UCSF Chimera 1.8.1^19^.

### Protein production

The FADD sequence 1-191 was cloned in a modified pET21 vector with a C-terminal hexa-histidine tag and expressed in Rosetta-gami 2 DE3 pLysS cells at 32 °C overnight. Cells were lysed using a French Press and the whole lysate was denatured in 8 M urea. After centrifugation, the supernatant was applied to a HisTrap column (GE Healthcare) and the protein was refolded on column. The protein was then concentrated and purified by size exclusion chromatography (SEC) using a Superdex75 16/60 column (GE Healthcare).

The DNA for the GST-caspase 8 fusion protein was a gift from Dr. M. E. Peter (Northwestern University). The 80 kDa protein was expressed in the E. coli strain BL21 (DE3). Cultures were grown at 37 °C in Terrific Broth, induced with 0.2 mM IPTG and transferred to 20 °C for 16-18 hours. The protein formed inclusion bodies, which were washed four consecutive times in buffer containing either 2% Deoxycholate or 0.2% Triton X-100, then washed twice in buffer without surfactants. Finally, the clean inclusion bodies were resolubilized in 8 M Urea, 50 mM Tris-HCl, pH = 8.0 and the protein was refolded through successive rounds of dialysis in the presence of additives (2 mM DTT, 10% glycerol, 100 mM L-Arginine).

cFLIP DNA was cloned into pET21b+, and the protein was expressed with a C-terminal hexa-histidine tag. The protein expressed in the Rosetta (DE3) pLysS strain after induction with 0.2 mM IPTG and overnight incubation at 32 °C. The protein was resolubilized out of the pellet in 8 M urea, 50 mM Tris-HCl, 500 mM NaCl, pH = 8.0 and purified on Ni-NTA resin under denaturing conditions. Refolding was achieved through successive rounds of dialysis in 50 mM Tris-HCl, 150 mM NaCl, 5% glycerol, 0.2% NP40, 1 mM DTT, pH = 8.0.

The cytoplasmic domain of DR5 was cloned into pGEX-4T1 between the BamHI and EcoRI restriction sites. The GST-fusion protein was expressed in E. Coli BL21 (DE3) as described above and purified using a GSTrap column (GE Healthcare) followed by SEC on a Superdex 75 16/60 column (GE Healthcare).

### STD-NMR binding analysis

NMR experiments were carried out on a Bruker 700 MHz spectrometer equipped with a TCI cryoprobe, at 25 °C. The STD-NMR experiments^19^ utilized water suppression by excitation sculpting^20^ and interleaved acquisition of on- and off-resonance spectra (saturation at 0 Hz and −2000 Hz respectively). Saturation was achieved by a cascade of 50 ms Gaussian pulses for a period of 3 sec. A spin-lock period of 15 ms was applied to suppress residual protein resonances. 32k points and 256 transients were acquired for each experiment. Lorentzian broadening (0.3 Hz) was applied before Fourier transform.

Each sample contained the target protein (2 μM for caspase 8 or cFLIP, 10 μM for DR5, 5 μM for FADD, 3 μM for the GST-tag) and 200 μM of the small molecule tested. The caspase 8 samples utilized 50 mM phosphate buffer, pH 7.5, 250 mM NaCl, 5% D_2_O (deuterium oxide).

Saturation transfer from the protein to the bound small molecules causes a decrease in the intensity of the small molecule resonances, which is detected as residual signal in a difference spectrum.

### Cell lines and cell culture reagents

K562, Jurkat and PC3 cell lines were purchased from ATCC. These cell lines were grown in RPMI 1640 medium (Mediatech Inc., VA, USA, Cat. # 10-040-CV) supplemented with 10% fetal bovine serum (FBS, HyClone, Cat. # SH30088.03), 2 mM L-glutamine (Mediatech, Cat. # 25-005-CI) and 100 units/ml Penicillin/ 100 μg/ml Streptomycin (Mediatech Inc., Cat. # 30-001-CI).

HeLa cells were obtained from the ATCC and cultured in DMEM (Mediatech Inc., VA, USA) supplemented with 10% fetal bovine serum, 5 mM L-glutamine and 100 units/ml Penicillin/ 100 μg/ml Streptomycin. HeLa cells stably expressing combinations of IC-RP (caspase 8 activity reporter), and IMS-RP (Mitochondrial Outer Membrane Permeabilization (MOMP) reporter) were derived by infection with Adenovirus, followed by double positive selection by flow cytometric analysis. Sorted single cells were grown in 96-well plates until apparition of colonies. Cells were passed every 2-3 days and used in experiments during their logarithmic growth phase.

### Reagents

Compound 1 (3-Morpholin-4-yl-1-[5-(3-nitro-phenyl)-3-phenyl-4,5-dihydro-pyrazol-1-yl]-propan-1-one), Compound 2 (3-Cyclopropyl-6-[4-(2-phenyl-ethenesulfonyl)-piperazin-1-yl]-[1,2,4]triazolo[4,3-b]pyridazine) and Compound 3 (1-(2-Indol-1-yl-acetyl)-piperidine-4-carboxylic acid (5-methyl-isoxazol-3-yl)-amide) were provided by Spoluka Chemical Company (aka Life Chemicals, Inc., Kiev, Ukraine). Compound 4 (1-Azaxanthone; 5H-chromeno[2,3-b]pyridin-5-one) was provided by Maybridge (Cornwall, England). The compounds were used at 20, 40 or 80 μM (initial testing was done with concentrations ranging between 5 μM-320 μM).

Recombinant human (rh) TRAIL was a kind gift of Dr. Carlos Reis (University of Groningen, the Netherlands)^21^,^22^. TRAIL was used at 7.5 ng/ml (Jurkat), 10 ng/ml (PC3) and 45-100 ng/ml (K562).

Suspension cells (K562 and Jurkat) were counted and plated at specific concentrations in 24 well plates or 6 well plates, followed by the treatment with DMSO, TRAIL, the compounds and the combination for 8-28 h in 0.5-1% FBS (see the figure legends for specific details).

### Reporter constructs

Caspase 8 activity reporter (IC-RP) and Mitochondrial Outer Membrane Permeabilization (MOMP) reporter (IMS-RP) were constructed as previously described^23^.

### Viability / cell death analysis (automated trypan blue exclusion assay)

Viability and cell death was quantified by using an automated Trypan blue exclusion method as previously described^24^. BioRad TC10 automated cell counter is designed to count Trypan blue positive and negative suspended cells and can determine viability by an automated image analysis algorithm with high reproducibility when the concentration of cells is within 5 × 10^4^–10^7^ cells/ml. Briefly, after collection of the suspension (K562 and Jurkat) or adherent cells (PC3), a volume of 30-40 μl of cells was mixed with an equal volume of Trypan Blue solution (0.4%). 10 μl of this mix was then loaded on a special counting chamber and read with the BioRad TC10 cell counter after 10 seconds. For adherent cells, PC3 cells were washed with DPBS (Mediatech, Inc, Cat. # 21-031-CV) and collected by using Trypsin EDTA (0.25% Trypsin / 2.21 mM EDTA in HBSS; Mediatech, Inc., Cat. # 25-053-CI). Each experiment was repeated three times, except if indicated otherwise. For every experiment, each treatment was performed in duplicate and multiple readings were performed for each condition.

### Western blotting

Cells were washed with PBS and lysed in RIPA buffer (Boston BioProducts, Boston, USA, Cat. # BP-115D). SDS-PAGE and the immunoblotting were performed as previously described^25^,^26^. Whole cell lysates (30 μg) in reducing Laemmli’s SDS Sample Buffer (Boston Bioproducts, Cat # BP-110R) were resolved by 10% SDS-PAGE, and transferred to 0.45 μm Immobilon PVDF membranes (Millipore, MA, USA Cat # IPVH00010). The membranes were then blocked for 30 min at room temperature in blocking buffer (5% w/v non-fat dry milk/Tris-buffered saline-Tween-20) (TBST: 50 mM Tris-HCl (pH 7.4), 150 mM NaCl, 0.05% (v/v) Tween-20). Rabbit/mouse primary antibodies were diluted in 5% (w/v) blocking buffer then incubated with membranes overnight at 4 °C, or for 2 h at room temperature. Membranes were then washed three times with TBST, incubated for 1 h at room temperature with either HRP-conjugated goat anti-mouse or goat anti-rabbit IgG secondary antibodies (sc-2055 and sc-2054, respectively, Santa Cruz Biotechnology, CA, USA) diluted 1: 3500-4000 in blocking buffer, and then washed again with TBST prior to visualization using a chemiluminescent substrate (Pierce ECL Western Blotting Substrate, Super Signal West Pico or Super Signal West Femto, Pierce - Thermo Scientific, IL, USA).

Most of the antibodies employed for probing the Immobilon PVDF membranes were purchased from Cell Signaling Technology (MA, USA) and used at dilution of 1:1,000-1,500: PARP (# 9542), caspase 3 (# 9662), cleaved caspase 3 (# 9664), caspase 8 (# 9746), cleaved caspase 8 (# 9496), caspase 9 (# 9502), IKappaBα (# 9242). Anti-β-Actin antibody (clone AC-15) (# A5441) was purchased from Sigma (MO, USA) and used at a dilution of 1:20,000.

### Photomicrograph and image processing

Phase contrast / bright field microscope images were taken using a Nikon Eclipse TI-S Inverted Microscope (4x or 10x objective lenses) and by using QCapture 2.99.5 software (2009, Quantitative Imaging Corporation).

### Live-Cell Microscopy and image analysis of single cells

Time-lapse microscopy movies were recorded with a Deltavision fluorescence microscope equipped with an environmental chamber (Olympus, Applied Precision) at 10x magnification with frames every 3 minutes for 24 hours. Cells grown in 8-well chambered cover glass slides (Nunc) were shifted into phenol red-free medium (Invitrogen) supplemented with 10% fetal bovine serum and L-glutamine for imaging. For FRET signal analysis, ratio of background-subtracted CFP and YFP images were created by using ImageJ and custom plug-ins^23^. Signals were normalized by subtracting the minimum value across all time points from each single-cell time course. IMS-RP release in cell cytoplasm was analyzed in ImageJ by visual inspection, which enabled an identification of the first frame of IMS-RFP release and subsequent time of cell death of each single-cell of the cell population analyzed.

## Results

### In silico identification of the small molecules that bind caspase 8 and experimentally sensitize Jurkat cells to TRAIL-induced apoptosis

Small molecules that can activate caspase 8 and/or sensitize resistant cells to death ligands-induced cell death have the potential to become an exciting class of novel pro-apoptotic cancer therapies. We designed an *in silico* screening to identify for the first time small molecules that bind caspase 8 and stabilize the zymogen caspase 8 homodimerization, resulting in a productive caspase 8 conformation that initiates faster processing of mature caspase 8.

**Figure 1:**
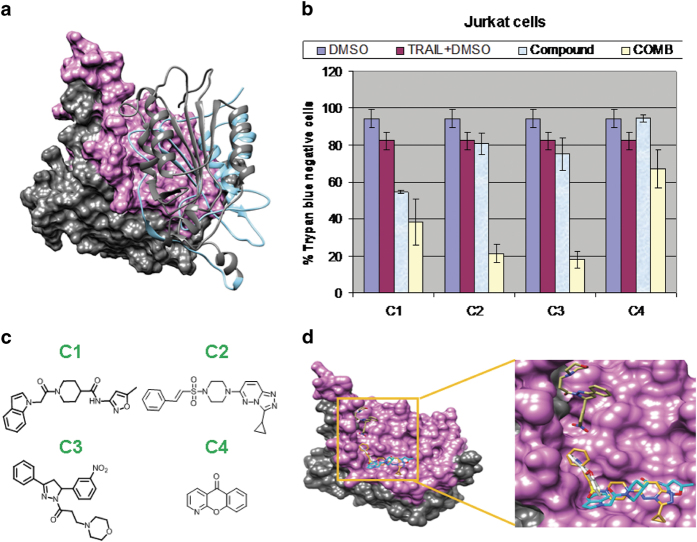
*In silico* discovery of small molecules that bind caspase 8 and their experimental effect on Jurkat cells survival; Zymogen caspase 8 interface region containing the small subunit (purple) was targeted and zymogen caspase 8 model (based on (PDB ID: 3H13)) was used as the receptor for *in silico* molecular docking studies. (**a**) Zymogen caspase 8 homodimer showing the interface region with one caspase 8 monomer in surface representation and the other monomer in ribbon view. Different domains of the caspase 8 are distinguished by color: large subunits are shown in grey and small subunits shown in purple and light blue. A grid box was introduced spanning whole interface region for the search space. (**b**) Compounds that experimentally sensitize Jurkat cells to TRAIL-induced apoptosis. Cell death/viability was evaluated in Jurkat cells by using an automated Trypan blue exclusion essay. Jurkat cells were treated with DMSO (control), TRAIL 7.5 ng/ml, the compounds (80 μM) and the combination, for 24 h, in 1% FBS. Results show that all four compounds sensitize Jurkat cells to TRAIL-induced cell death, with compounds 2 and 3 being the most effective. First compound can also induce significant cell death when used alone. (**c**) The 2D structures of the hit compounds 1-4, which were experimentally validated to sensitize Jurkat cells to TRAIL-induced cell death. (**d**) 3D conformation of the hit compounds bound to caspase 8 interface region. Note: compound 1 (cyan), compound 2 (gold), compound 3 Khaki and compound 4 (white). Right panel: enlarged boxed area of the left panel; C1 = compound 1; C2 = compound 2; C3 = compound 3; C4 = compound 4.

We targeted the zymogen caspase 8 interface region formed by the small subunit (purple, Fig. 1A): by first generating a model of the zymogen caspase 8 structure based on the X-ray structure of zymogen caspase 8 (lacks L2 loop region) in complex with cFLIP_L_ (PDB ID: 3H13) and then using it for *in silico* molecular docking studies. The top four compounds resulting from the *in silico* screening (labeled as C1-C4 in Fig. 1B,C) were then experimentally tested in Jurkat cells for their potential to induce cell death alone or in combination with the death ligand Apo2L/TRAIL (Fig. 1B). Jurkat cells were treated with DMSO (control), TRAIL, compounds 1-4 and the combination, for 24 h. Results show that all four compounds sensitize Jurkat cells to TRAIL-induced cell death, with compounds 2 and 3 being the most effective. Compound 1 can also induce significant cell death when used alone (Fig. 1C). A predicted 3D conformation of the hit compounds bound to the caspase 8 interface region, as derived from the docking effort, is depicted in Fig. 1D.

### Small molecule compound 2 directly binds caspase 8

**Figure 2:**
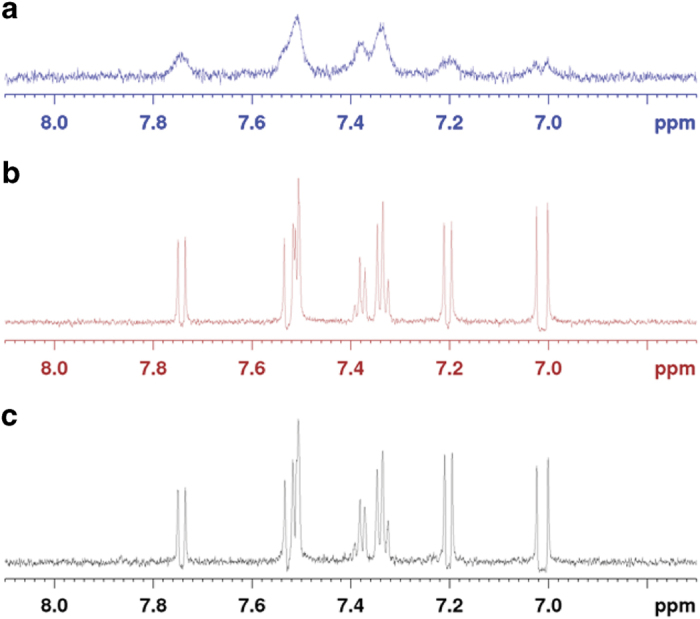
Compound 2 directly binds caspase 8. Small molecule compound 2 directly binds caspase 8, as determined by NMR. (**a**) extensive line broadening of compound 2 resonances caused by binding to the high molecular weight caspase 8. Line broadening is not observed in the control experiments in the presence of GST (**b**) or buffer alone (**c**).

In order to determine if the four compounds that sensitize Jurkat cells to TRAIL-induced cell death are indeed directly binding caspase 8, we performed saturation transfer difference (STD)-NMR experiments (Fig. 2). We tested the molecules for binding to caspase 8 and three additional components of the DISC as a control: Death Receptor 5 (DR5), cFLIP and FADD. The NMR spectrum of compound 2 in the presence of caspase 8 shows pronounced line broadening due to the direct interaction with the high molecular weight caspase 8 and slower tumbling in solution (Fig. 2A). No line broadening was observed in the control experiments in presence of the GST fusion protein alone (Fig. 2B), or buffer alone (Fig. 2C). Compound 1 and compound 4 display no line broadening and a small residual signal in STD-NMR experiments, evidence of very weak binding to caspase 8. Compound 3 does not show binding to caspase 8, within the affinity ranged sampled by STD-NMR (μM-mM). In our STD-NMR binding experiments we also observed that compounds 1, 3 and 4 weakly bind the DR5 receptor (Supplementary Figure 1). Noteworthy, compound 2 not only is the best binder for caspase 8, but, as shown below, together with compound 3, it is also the most effective compound in sensitizing Jurkat cancer cell line to TRAIL-induced cell death. Thus, our following experiments mainly focused on compound 2.

### Small molecule compound 2 (CaspPro) is an activator of caspase 8, potentiating TRAIL-induced caspase 8 activation in single cells and populations of cells

**Figure 3:**
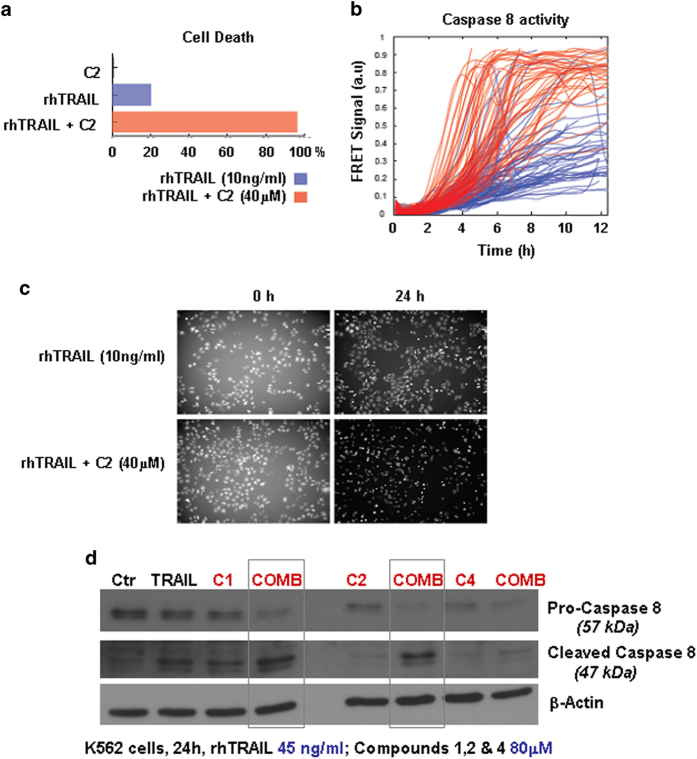
Compound 2 (CaspPro) potentiates TRAIL-induced Caspase 8 activation in *single cells* (a-c) and in populations of cells (d). **(a-c)** FRET detection of caspase 8 activation in a time-lapse experiment within *single living HeLa cells*. Caspase 8 is activated faster and at higher levels by TRAIL when CaspPro (40 μM) is present, resulting in greater cell death. (**d**) K562 leukemic cells were treated with DMSO (control), 45 ng/ml TRAIL, 80 μM compounds 1, CaspPro and 4, and the combination, for 24 h, in 0.5% FBS, followed by western blot detection of the pro-caspase 8 and cleaved caspase 8. While 80 μM CaspPro alone can’t induce an increase in the cleaved caspase 8 (active caspase 8), CaspPro in combination with TRAIL increases TRAIL-induced caspase 8 activation. 80 μM compound 1 is able to induce some caspase 8 cleavage alone and can also increase the TRAIL-induced caspase 8 activation. 80 μM compound 4 does not have any effect on caspase 8 activation when used alone, and does not potentiate TRAIL-induced caspase 8 activation; Cleaved Caspase 8, Caspase 8 and β-Actin were detected on the same membrane, after stripping and reprobing the membrane. The blot images were cropped to show the proteins of interest; C1 = compound 1; C2 = compound 2 (CaspPro); C4 = compound 4.

To further analyze if small molecule compound 2 can activate caspase 8 alone or in combination with TRAIL, we monitored caspase 8 activity by live cell microscopy, using a caspase 8 specific FRET reporter. We found that caspase 8 was activated faster and at higher levels when cells were stimulated by a combination of TRAIL and compound 2. Consequently, co-treatments with TRAIL and compound 2 together also resulted in greater cell death (96%), compared to TRAIL or compound 2 alone (20% and 1% respectively) (Fig. 3A–C). These results were subsequently confirmed in population of cells, by performing western blotting on the whole cell lysate. While activation of caspase 8 as measured by zymogen caspase 8 cleavage is not seen when cells are treated with compound 2 alone, compound 2 significantly promotes caspase 8 activation induced by TRAIL (Fig. 3D). Thus, this small molecule was named CaspPro. By using Jurkat leukemic cell lines deficient in caspase 8, we could also confirm that caspase 8 is required for CaspPro-mediated potentiation of TRAIL-induced cell death (Supplementary Figure 2).

### Small molecule CaspPro sensitizes K562 leukemic cells to TRAIL-induced caspase 3 activation and cell death

**Figure 4:**
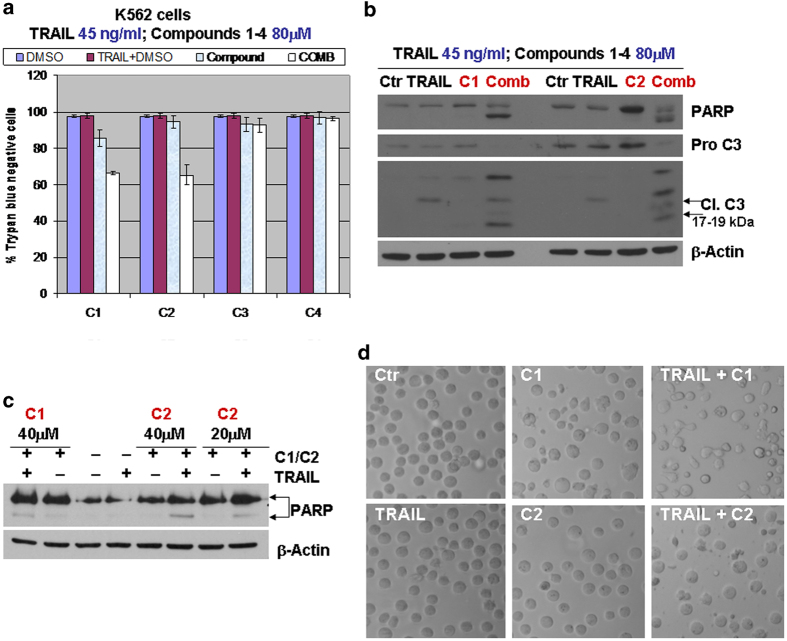
Compounds 2 (CaspPro) and 1 sensitize leukemic K562 cells to TRAIL-induced apoptosis. **(a)** K562 leukemic cells were treated with DMSO (control), 45 ng/ml TRAIL, 80 μM compounds 1-4, and the combination, for 24 h, in 0.5% FBS. Viability was measured by an automated Trypan Blue dye exclusion method. Results represent the mean +/- standard deviations (SDs) of 3 independent experiments, each one with multiple replicates. The results demonstrate that only compounds 1 and 2 are effective in sensitizing K562 cells to TRAIL-induced cell death, while compounds 3 and 4 do not show any effect at 80 μM. (**b**) K562 cells were treated with DMSO, 45 ng/ml TRAIL, 80 μM compounds 1 and 2, and the combination, for 24 h, followed by detection by western blot of the pro-caspase 3, cleaved caspase 3 and PARP cleavage (caspase mediated). The combined regimen resulted in pro-caspase 3 and PARP cleavage, implying caspase 3 activation. (**c.**) K562 cells were treated with DMSO, 28 ng/ml TRAIL, 20 μM or 40 μM CaspPro, 40 μM compound 1, and the combination, for 24 h, followed by detection of the PARP cleavage. CaspPro at 20 μM and 40 μM, and compound 1 at 40 μM, sensitize K562 to TRAIL-induced PARP cleavage, indicative of caspase activation. (**d**) K562 leukemic cells were treated with DMSO, 45 ng/ml TRAIL, 80 μM compounds 1 and 2, and the combination, for 24 h. Phase contrast microscope images of the untreated or treated cells are presented. A Nikon Eclipse TI-S Inverted Microscope was used (10X objective lens). While single treatments did not significantly change the morphology and number of the cells (except in the case of 80 μM compound 1, where some cells have apoptotic-like features), the combined treatment induced morphological changes and a decrease cell number. β-Actin was used as an internal loading control. A total of three independent experiments were performed for each panel. All the proteins presented in a single panel (see b or c) were detected on the same membrane, by stripping and re-probing with a different antibody. The images of each membrane were cropped for showing the proteins of interest; experiments were done in 0.5% FBS; C1-4 = compounds 1-4.

To further validate the effect of CaspPro in sensitizing cancer cells to TRAIL-induced cell death, we tested the compound in K562. This Bcr-Abl positive leukemic cell line is a highly resistant cell line to TRAIL action^27^. The underlying mechanisms of tumors cells resistance to TRAIL are not fully elucidated. Resistance can occur due to the dysregulation/deregulation of apoptotic signaling pathways, including the lack or decrease expression of the death receptors DR4 and DR5, and dysregulation of either pro-apoptotic or anti-apoptotic factors^27^. Our results demonstrate that only compounds 1 and 2 are effective in sensitizing K562 cells to TRAIL-induced cell death, while compounds 3 and 4 do not show any effect. Interestingly, CaspPro is the most effective compound in sensitizing K562 resistant cells to TRAIL-induced cell death (Fig. 4A).

Furthermore, we determined that the combined regimens (compounds 1 or 2 in combination with TRAIL) resulted in pro-caspase 3 and PARP cleavage, implying apoptosis activation (Fig. 4B,C). While single treatments did not significantly change the morphology and number of the cells (except in the case of compound 1 when used alone, where some cells have apoptotic-like features), the combined treatment induced a change in morphology/shape and a decrease in the number of the cells (Fig. 4D).

### Small molecule CaspPro sensitizes PC3 prostate cancer cells to TRAIL-induced caspase 3 activation and cell death

**Figure 5:**
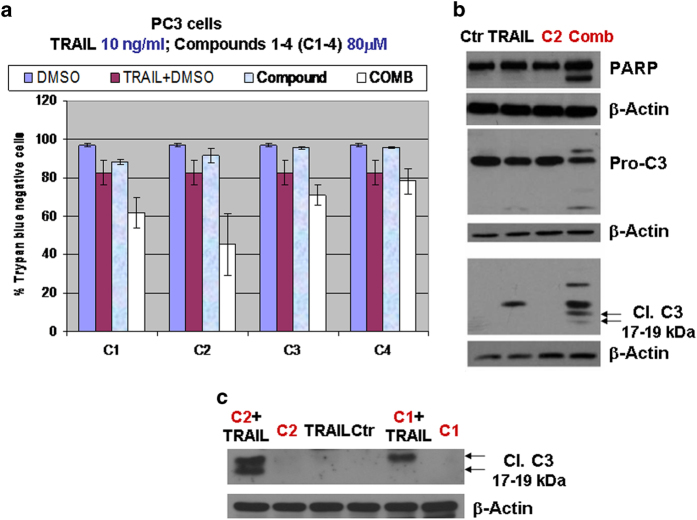
Compounds 1, 2 (CaspPro), 3 and 4 sensitize prostate cancer cells to TRAIL-induced apoptosis **(a)** PC3 prostate cancer cells were treated with DMSO (control), 10 ng/ml TRAIL, 80 μM Compounds 1-4 and the combination, for 24 h, in 0.5% FBS. Viability was measured by Trypan Blue dye exclusion method, using a TC10 Automated Cell Counter (Biorad, USA). The results represent the mean +/- standard deviations (SDs) of 3 independent experiments, each one with multiple replicates. Similar to the results obtained in K562 cells, compounds 1 and 2 are the most effective in sensitizing PC3 cells to TRAIL-induced cell death, while the effect observed with compounds 3 and 4 is smaller. (**b**) PC3 cells were treated with DMSO (control), 10 ng/ml TRAIL, 80 μM CaspPro, and the combination, for 24 h, in 0.5% FBS, followed by detection by western blot of the pro-caspase 3, cleaved caspase 3 and PARP cleavage. The combined regimen resulted in pro-caspase and PARP cleavage, suggesting caspase 3 activation. This experiment was performed 3 times with similar results. (**c.**) PC3 cells were treated with DMSO (control), 10 ng/ml TRAIL, 40 μM compounds 1 and 2, and the combination, for 24 h, in 0.5% FBS, followed by detection of the cleaved caspase 3 by western blot. Both compounds 1 and 2 sensitize PC3 cells to TRAIL-induced caspase 3 activation. The blots presented in B and C were cropped and the proteins of interest are presented. Each membrane was stripped and re-probed with an antibody anti β-Actin, which is used as an internal loading control. The gels have been run under the same experimental conditions; C1-4 = compounds 1-4.

Our results were further validated in PC3 prostate cancer cell line. Similar to the results obtained in K562 cells, CaspPro is the most effective in sensitizing PC3 cells to TRAIL-induced cell death, followed by compound 1, while the effects observed with compounds 3 and 4 are smaller (Fig. 5A). Moreover, CaspPro sensitizes PC3 cells to TRAIL-induced caspase 3 activation and PARP cleavage (Fig. 5B), results also observed with compound 1 (Fig. 5C).

### CaspPro can stabilize the caspase 8 homodimer interface

**Figure 6:**
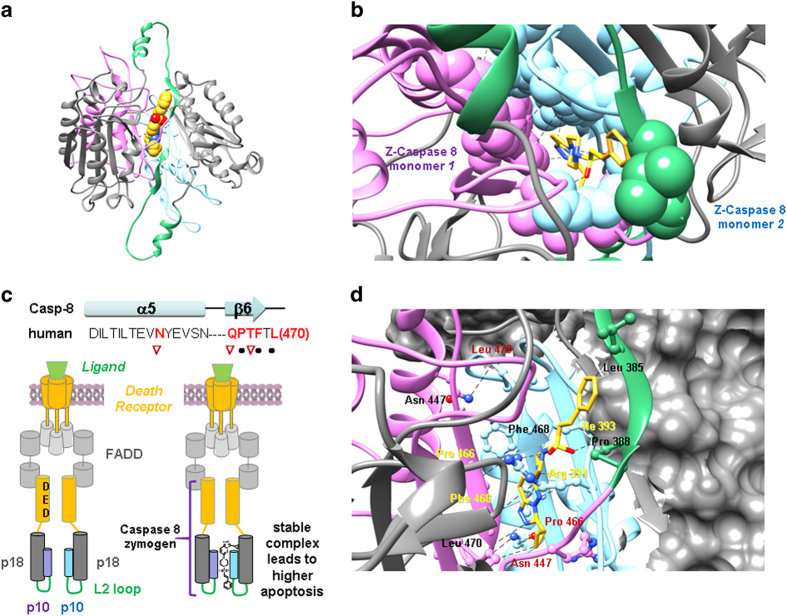
Model for CaspPro binding to zymogen caspase 8 homodimer **(a)** CaspPro is shown as spheres in the central cavity at the dimer interface of zymogen caspase 8 (ribbon view). The large subunit (p18, grey), small subunit (p10, light blue and purple) and L2 loop (green) are shown. In our model CaspPro favors zymogen caspase 8 homodimerization that results in a productive conformation that initiates faster processing of mature caspase-8 when death receptors are engaged. (**b**) Residues involved in forming contacts in the binding region for CaspPro (sticks, yellow) are shown as spheres. (**c**) Sequence of caspase 8 involved in homodimerization at the β6 interface. Conserved residues that formed H bonds between the monomers are shown in red. Residues involved in main-chain (black circles) and side-chain (red triangles) interactions are shown. The schematic drawing of the proposed caspase 8 activation mechanism is shown in the lower panel, where CaspPro provides additional stabilization of the caspase 8 homodimer. (**d**) Putative binding sites of CaspPro in the model structure of zymogen caspase 8 homodimer. Side-chains of residues likely involved in the mechanism of activation of zymogen caspase 8 by CaspPro are displayed in ball and stick view. These include H-bonds, ionic and Van der Waals interactions that may be necessary for stabilization and activity of the zymogen caspase 8 homodimerization. Large subunit of one monomer is shown in surface view for clarity. Z-Caspase 8 = zymogen caspase 8.

Using the experimentally determined structure of the caspase 8/cFLIP dimer (PDB 3H11), we created a model of the caspase 8 homodimer (Fig. 6A). We observed that including CaspPro in the preferred orientation while bound to monomer 1, makes a number of favorable contacts with monomer 2, and thereby stabilizes the formation of the dimer. Specifically, the aromatic ring of CaspPro is within a hydrophobic pocket formed by L385, L 393, and F468 of monomer 2. Additionally, we propose hydrogen bonds between the sulfoxide oxygens of CaspPro and backbone amides of monomer 2. All of this interactions support the stabilization of the dimer.

## Discussion

*In silico* screening has been used as a successful approach in finding *inhibitors* for several targets implicated in different pathologies, including cancer^28^. These include the discovery of BRD4 (belongs to bromodomain family) inhibitors as therapeutics for acute myeloid leukemia, other malignancies and inflammatory diseases. Crystal structures of the *in silico* hits were later solved confirming their binding mode^29^.

Our findings indicate that *in silico* screening can also be successfully used to identify small molecule *activators* of different targets, such as caspases, highlighting the importance of *in silico* screening as a vital aid in the initial drug discovery efforts of both inhibitors and activators small molecules. A recent study points out the identification of small molecules activators of the effector caspases 3 and 7, which act by increasing their zymogen’s proteolysis^30^. However, these small molecules are not direct activators of caspases, as demonstrated by a subsequent study^31^. CaspPro directly binds caspase 8 and potentiates TRAIL-induced caspase 8 activation in single cells and populations of cells, event followed by activation of caspase 3 and induction of cell death. A schematic representation of our findings and a proposed caspase 8 activation mechanism where CaspPro provides additional stabilization of the caspase 8 homodimer is shown in Fig. 6.

The pocket at the caspase dimerization interface of caspases has been successfully targeted to identify novel inhibitors^14^,^15^. These inhibitors suppress the caspase activation and apoptotic process. However, identification of small molecules that can stabilize the zymogen caspase 8 homodimerization and promote caspase 8 activation has a great potential for use in cancer therapy. Dimerized uncleavable chimeric caspase 8 constructs can be productively activated *in vitro*^32^. Other studies have shown that recombinant caspase 8 containing the large and small subunits can be induced to dimerize by the addition of kosmotropic salts (e.g. 1 M sodium citrate), which maximally activates caspase 8^33^,^34^. However, to our knowledge, small molecules that can stabilize the zymogen caspase 8 homodimerization and promote activation of caspase 8 when death receptors are activated have not been developed.

In our proposed model, CaspPro favors zymogen caspase 8 homodimerization that results in a productive conformation initiating faster processing of mature caspase 8 when death receptors are engaged (Fig. 6A,B). CaspPro may provide additional stabilization of the caspase 8 homodimer which promotes caspase 8 activation after TRAIL treatment, leading to caspase-dependent cell death (Fig. 6C). Thus, although CaspPro alone can’t induce caspase 8 activation, it can significantly potentiate TRAIL-induced caspase 8 activation and cell death. The putative binding sites of CaspPro in the model structure of zymogen caspase 8 homodimer are shown in Fig. 6D. Additional experiments need to be performed to validate these CaspPro - zymogen caspase 8 putative binding sites in the future.

Our experimental validation results performed in multiple cell lines, such as leukemic Jurkat and K562 cells, and prostate PC3 cell line, revealed that CaspPro promotes caspase 8 activation, caspase 3 activation and PARP cleavage, in the presence of TRAIL, leading to cell death. The development of methods for analyzing single living cell activity and signaling is critical for the advancement of a wide variety of biomedical fields. By using a FRET detection of caspase 8 activation in a time-lapse experiment within *single living* cells, we were able to show that CaspPro promotes earlier and higher caspase 8 activation in single cells after TRAIL treatment, compared to TRAIL treatment alone.

Recent studies have shown that approximately 60% of cancer cell lines are resistant to TRAIL. Thus, sensitizing cancer cells to TRAIL-induced cell death is necessary^35^,^36^. We have shown that CaspPro can sensitize three different cancer cell lines (leukemic Jurkat and K562 cell lines, and prostate PC3 cell lines) to TRAIL-induced cell death. However, in order to obtain a comprehensive evaluation on CaspPro potential many more cancer cell lines and cancer mouse models have to be employed. The use of mouse models will also make possible CasPro’s safety profile evaluation.

Our findings establish a proof of concept strategy for discovery of novel small molecule activators or inhibitors of DISC related proteins, and a foundation for future development of caspase 8-selective small molecules that promote caspase 8 activation in response to death receptors stimulation.

## Author Contributions

O.B., G.G., A.Y., B.P., C.R., D.M. and R.K.-F. contributed to the conception and design of the study; O.B., G.G., A.Y., B.P., C.R., J.R., M.P., D.M. & R.K.-F. helped in the development of methodology; A.Y., C.R., G.G., M.P., A.P. and D.M. helped with the in vitro screening and binding validation by N.M.R.; O.B., B.P., J.R., S.A. and B.G. helped with the characterization and validation of the small molecules; JR did the single cell analysis; all authors significantly contributed to the study and to the writing of the manuscript; D.M. and R.K.-F. supervised the study;

## Additional Information

**How to cite this article**: Bucur, O. *et al.* A novel caspase 8 selective small molecule potentiates TRAIL-induced cell death. *Sci. Rep.* **5**, 9893; doi: 10.1038/srep09893 (2015).

## Supplementary Material

Supplementary Information
