# Supplementary material for: A novel caspase 8 selective small molecule potentiates TRAIL-induced cell death
Source: Sci Rep. 2015 May 11;5:9893. doi: 10.1038/srep09893 (PMC4426715; doi:10.1038/srep09893)
Supplement: Supplementary Information [file srep09893-s1.pdf]

Supplementary Figures

A novel caspase 8 selective small molecule potentiates TRAIL-induced cell death

Octavian Bucur[1,4,]*, Gabriel Gaidos[2,]*, Achani Yatawara[2,]*, Bodvael Pennarun[1], Chamila Rupasinghe[2], Jérémie Roux[3], Stefan Andrei[1], Bingqian Guo[2], Alexandra Panaitiu[2], Maria Pellegrini[2], Dale F. Mierke[2#], Roya Khosravi-Far[1,5#]

[1]Department of Pathology, Beth Israel Deaconess Medical Center and Harvard Medical School, Boston, MA, USA;
[2]Department of Chemistry, Dartmouth College, Hanover, NH, USA;
[3]Department of Systems Biology, Harvard Medical School, Boston, MA, USA;
[4]Institute of Biochemistry of the Romanian Academy, Bucharest, Romania;
[5]Department of Biological and Biomedical Sciences Program, Harvard Medical School, Boston, MA, USA

*These authors contributed equally to this work
#Corresponding authors:
Roya Khosravi-Far, PhD, Department of Pathology, Beth Israel Deaconess Medical Center and Harvard Medical School, 330 Brookline Avenue, Boston, MA 02215, USA; E-mail: rkhosrav@gmail.com
Dale F. Mierke, PhD, Chair, Department of Chemistry, Dartmouth College, 6128 Burke Hall, Hanover, NH 03755, USA. E-mail: dale.mierke@dartmouth.edu

A

STD-NMR binding results

Target	Compound 1	Compound 2	Compound 3	Compound 4
	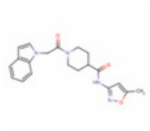	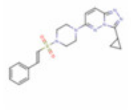	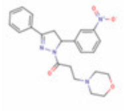	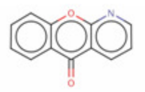
**Caspase 8**	+ v. small	**broadening** (see Figure 2)	-	-
**cFLIP**	-	-	-	-
**DR5**	**+ small** (see Suppl. Fig 1B)	-	+ v.v. small	**+ small** (see Suppl. Fig 1C)
**FADD 4.8μM**	-	-	-	N/A
**FADD 5.7μM**	-	+ v.v. small	-	+ almost nothing
**GST (Ctrl)**	-	-	N/A	-

B

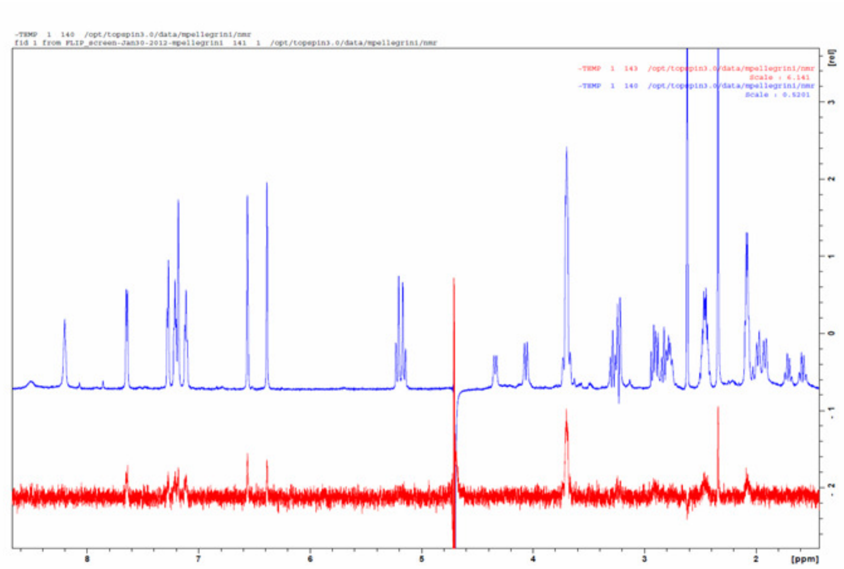

DR5 + C1

Blue: reference spectrum, C1 + DR5

Red: STD difference spectrum

C

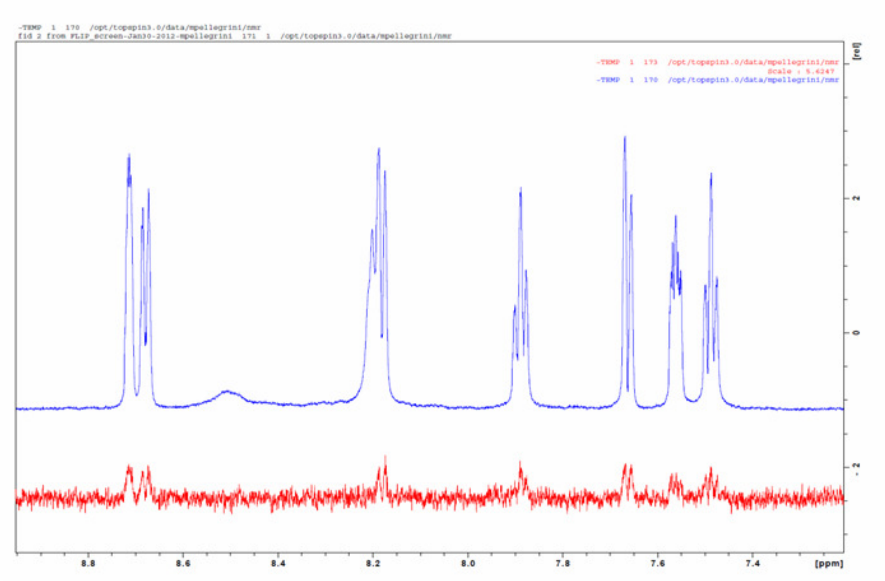

DR5 + C4

Blue: reference spectrum, C4 + DR5

Red: STD difference spectrum

Supplementary Figure 1. Binding potential of C1-C4 small molecules to Caspase 8, DR5, FADD and cFLIP

A. A summary of the binding potential of C1-C4 compounds to Caspase 8, DR5, FADD and cFLIP is presented. STD-NMR experiments were performed as described in the Material and Methods section of the manuscript. Saturation transfer from the protein to the bound small molecules causes a decrease in the intensity of the small molecule resonances, which is detected as residual signal in a difference spectrum. These experiments confirm that small molecule compound 2 directly binds caspase 8, as determined by the STD-NMR experiments, while compound 1 may also bind caspase 8, but with low affinity.

B.&C. Compounds C1 and C4 have some affinity towards binding DR5. Line broadening is not observed in the control experiments in the presence of GST or buffer alone;

"+ *v. small*" – very small broadening; "*v. v. small*" – very very small broadening; "-" – no broadening (no binding)

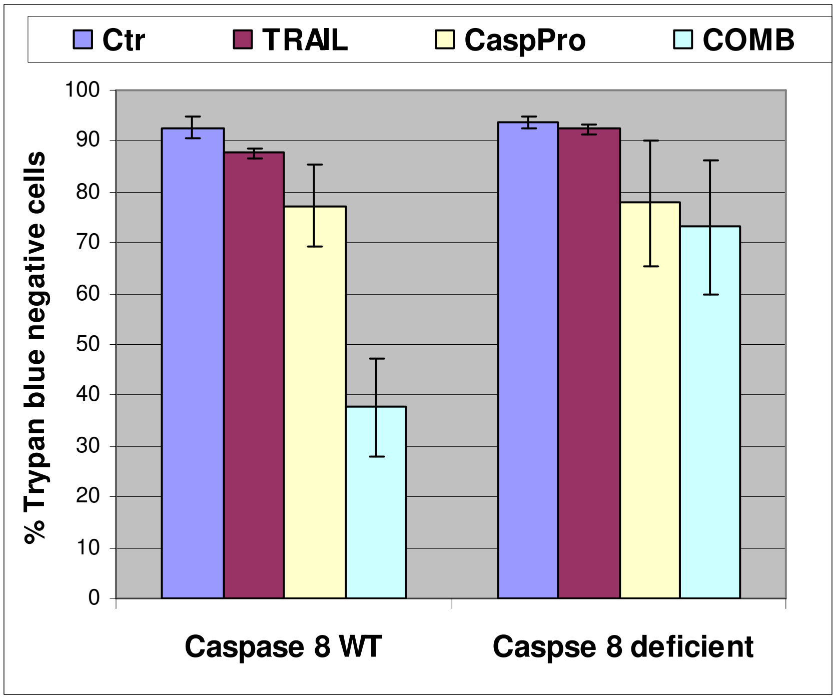

Supplementary Figure 2. Caspase 8 is required for CaspPro (C2, compound 2) - mediated potentiation of TRAIL-induced cell death

Caspase 8 WT and caspase 8 deficient Jurkat leukemic cells were treated with 7.5 ng/ml TRAIL, 80 µM CaspPro and the combination for 18h in 1% FBS. Viability was measured by Trypan Blue dye exclusion method, using a TC10 Automated Cell Counter (Biorad, USA). The results represent the mean +/- standard deviations (SDs) of 2 independent experiments, each one with multiple replicates. The results show that caspase 8 is required for CaspPro-mediated sensitization to TRAIL-induced cell death; We have previously confirmed the lack of expression of caspase 8 in caspase 8 deficient Jurkat cell line[8].
